# Supplementary material for: S-polarized light-sheets improve resolution and light-efficiency in oblique plane microscopy
Source: Sci Rep. 2024 Feb 12;14:3540. doi: 10.1038/s41598-024-53900-9 (PMC10861444; doi:10.1038/s41598-024-53900-9)
Supplement: Supplementary file 1 — Supplementary Information. [file 41598_2024_53900_MOESM1_ESM.pdf]

S-polarized light-sheets improve resolution and light-efficiency in oblique plane microscopy

Jon-Richard Sommernes[1], Alfred Millett-Sikking[2], Florian Ströhl[1,*]

[1]Department of Physics and Technology, UiT The Arctic University of Norway, Tromsø, Norway
[2]Calico Life Sciences LLC, South San Francisco, CA, USA
[*]Corresponding author: florian.strohl@uit.no

February 6, 2024

Tracing matrices

In all the simulations in this paper, we have used the Jones calculus to trace the electric field through the system. The Jones calculus is a matrix formalism that describes the polarization of light. For our case, we have modeled the system using the following matrices:

$$\mathbf{T}_{sys}(\theta_1, \phi, \alpha) = \mathbf{R}_{\mathbf{z}}^{-1}(\phi_\alpha)\mathbf{M}_3(\theta_3)\mathbf{R}_{\mathbf{y}}(\theta_3)\mathbf{F}_{\mathbf{T}}\mathbf{R}_{\mathbf{y}}(-\theta_2)\mathbf{R}_{\mathbf{z}}(\phi_\alpha)\mathbf{R}_{\mathbf{x}}(\alpha)\mathbf{R}_{\mathbf{z}}^{-1}(\phi)\overline{\mathbf{M}}_2(\theta_2)\mathbf{M}_1(\theta_1)\mathbf{R}_{\mathbf{z}}(\phi) \quad (1)$$

$$\mathbf{M}_i(\theta_i) = \mathbf{T}_i(\theta_i')\mathbf{O}_i(\theta_i)\Gamma_i(\theta_i)A_i(\theta_i) \quad , \quad \overline{\mathbf{M}}_i(\theta_i) = \mathbf{O}_i(\theta_i)\mathbf{T}_i(\theta_i')\Gamma_i(\theta_i)A_i(\theta_i) \quad (2)$$

All the matrices, except $\mathbf{F}_{\mathbf{T}}$, are simply rotation matrices. These matrices will map the electric field into a new coordinate system, without altering the amplitude of the field. In this section, we will describe the matrices and their role in the system.

The first matrix applied to the electric field is $\mathbf{R}_{\mathbf{z}}(\phi)$, and is defined as:

$$\mathbf{R}_{\mathbf{z}}(\phi) = \begin{bmatrix} \cos(\phi) & \sin(\phi) & 0 \\ -\sin(\phi) & \cos(\phi) & 0 \\ 0 & 0 & 1 \end{bmatrix} \quad (3)$$

where ϕ is the angle of rotation around the z-axis. This matrix is used to map the field from Cartesian coordinates into a the meridional and sagittal planes. This way, the electric field can eaily be traces through a lens using $\mathbf{L}(\theta)$, which is defined as:

$$\mathbf{L}(\theta) = \begin{bmatrix} \cos(\theta) & 0 & \sin(\theta) \\ 0 & 1 & 0 \\ -\sin(\theta) & 0 & \cos(\theta) \end{bmatrix} \quad (4)$$

where θ is the angle of the ray relative to the optical axis. Both $\mathbf{O}_(\theta)$ and $\mathbf{T}_(\theta)$ are identical to $\mathbf{L}(\theta)$, but distinguished for clarity to separate objectives and tube lenses. This matrix is used to simulate a lens, which is done by either collimating or focusing the electric field. The matrix $\mathbf{\Gamma}(\theta)$ is used to map the transmission of the lens, and is defined as:

$$\mathbf{\Gamma}(\theta) = \begin{bmatrix} T_p & 0 & 0 \\ 0 & T_s & 0 \\ 0 & 0 & 1 \end{bmatrix} \tag{5}$$

where T_p and T_s is the transmission of the lens for p- and s-polarized light respectively. In this paper, the transmission was measured experimentally. The next element is not a matrix, but simply a scalar function. This function, $A(\theta)$, is the apodization function of the lens. This can be found using the Abbe-Sine condition, and is given by:

$$A(\theta) = \sqrt{\frac{n}{\cos(\theta)}} \qquad A'(\theta) = \sqrt{\frac{\cos(\theta)}{n}} \tag{6}$$

where $A(\theta)$ is for a collimating lens, and $A'(\theta)$ is for a focusing lens. The next matrix is $\mathbf{R_x}(\alpha)$, which is used to rotate the electric field around the x-axis. In this paper, this matrix is used to simulate the rotation of the optical axis between O2 and O3. This matrix is given by:

$$\mathbf{R_x}(\alpha) = \begin{bmatrix} 1 & 0 & 0 \\ 0 & \cos(\alpha) & -\sin(\alpha) \\ 0 & \sin(\alpha) & \cos(\alpha) \end{bmatrix} \tag{7}$$

where α is the tilt of the optical axis. The next matrix is $\mathbf{R_y}(\theta)$, which is used to rotate the electric field around the y-axis. This is used to map the electric field from the meridional and sagittal planes into s- and p-polarized light. This matrix is given by:

$$\mathbf{R_y}(\theta) = \begin{bmatrix} \cos(\theta) & 0 & -\sin(\theta) \\ 0 & 1 & 0 \\ \sin(\theta) & 0 & \cos(\theta) \end{bmatrix} \tag{8}$$

where θ is the angle of the rays relative to the optical axis.

The last matrix is $\mathbf{F_T}$, which is a transmission matrix for a refractive index change. This matrix is identical in form to $\mathbf{\Gamma}(\theta)$. However, the transmissions for this matrix are calculated using the Fresnel equations. For our case, where the transmission through the refractive index change was measured along with the O3 transmission, this matrix was substituded with an identity matrix of equal size.

Sampling criterion

For the simulations to be valid, the electric field traced through the system needs to be properly sampled. For this to be true we need to sample fine enough for the phase to not change by more than π between two pixels. For this to be true, we can use the sampling condition outlined by Leutenegger et al.:

$$N_m > \frac{2\mathrm{NA}_f^2}{\sqrt{n_f^2 - \mathrm{NA}_f^2}} \frac{|z|}{\lambda_0} \tag{9}$$

where N_m is the minimum needed sampling points, n_f is the refractive index in the image space, z is the maximum axial distance from the focal point, NA_f is the numerical aperture of the focusing

lens, and λ_0 is the wavelength in vacuum. In our simulations, the sampling volume is a cube with isotropic resolution in the sample space. This means our $|z|$ can be found using:

$$|z| = \frac{vN_cM_a}{M_t} \tag{10}$$

where v is the voxel size, N_c is the number of voxels in the axial direction, M_a is the axial magnification of the system, and M_t is the transverse magnification of the system. Assuming a silicon immersion O_1, dry O_2, and a snouty O_3, we get $M_t = 56$ and $M_a = 2240$. Using a matched 0.025NA T_3 we get:

$$N_m \gtrsim 0.05\frac{v}{\lambda_0}N_c \tag{11}$$

We know that N_m must exist on the interval $N_m \in [0, N_c]$. As we will always sample with the full array of simulated voxels, we can set N_m to its maximum value N_c, leading to the condition $v \lesssim 20\lambda_0$. Assuming the sample should be Nyquist sampled, we also get the condition:

$$v \leq n_v < \frac{\lambda_0}{2\text{NA}_{O_1}}\frac{M_t}{2} \tag{12}$$

where n_v is the Nyquist voxel. Assuming a high NA O_1 (NA$\in (1, 1.4)$), we get the condition:

$$v < \frac{\lambda_0 M_t}{4\text{NA}_{O_1}} < 10\lambda_0 \tag{13}$$

Assuming Nyquist sampling, we can then conclude that our sampling rate is sufficient to avoid phase changes of more than π. The voxel size can also be determined using the equation outlined in[1]:

$$v = \frac{N_c\lambda_0}{N_p\text{NA}_f} \tag{14}$$

where N_p is the total number of pixels in one axis after padding the image to avoid aliasing. Redefining $N_p/2N_c = s$ where s is the scaling factor of the padding. To avoid aliasing, the scaling factor should be at least 2[1]. This results in the condition:

$$v = \frac{\lambda_0}{2s\text{NA}_f} < 10\lambda_0 \tag{15}$$

This means the requirement for our voxel size will always be met for a suitable scaling factor.

OTF background

To find the extent of an OTF, we need to find where the crossing from signal to backgound happens. To find this cutoff power, we use the assumed composition of our signal:

$$X = P(C_p) + G(\sigma_g) + b \tag{16}$$

$$X = C_p + \eta_p + \eta_g + b \tag{17}$$

where P is a poisson distribution of the photon count C_p resulting in noise η_p, G is gaussian noise of σ_g resulting in noise η_g, and b is a bias offset. The noise η_p and η_g are assumed to be independent and spectrally white. We can then find the variance of the signal using:

$$Var(X_i) = \cancelto{0}{Var(C_{p,i})} + Var(\eta_{p,i}) + Var(\eta_{g,i}) + \cancelto{0}{Var(b_i)} = \sigma_{p,i}^2 + \sigma_{g,i}^2 \tag{18}$$

where i is the pixel index. As the noise is spectrally white the variance for each pixel is independent. The variance of the entire signal is then given by:

$$Var(X) = \frac{\sum_i Var(X_i) + \cancelto{0}{\sum_{i \neq j} Cov(X_i, X_j)}}{N_c^3} = \overline{\sigma_p^2} + \overline{\sigma_g^2} \tag{19}$$

where $\overline{\sigma_p^2}$ and $\overline{\sigma_g^2}$ are the average variance of the poisson and gaussian noise respectively. We know that the variance of the poisson noise is equal to the mean, and the variance of the gaussian noise is given by the camera manufacturer by the electron RMS value. This means we can find the variance of the signal:

$$Var(X) = \overline{C_p} + \sigma_{RMS}^2 \tag{20}$$

where $\overline{C_p}$ is the average signal power without the bias offset. To correlate this variance to the OTF, we assume the signal can be decomposed as in equation 17. As the Fourier transform is a linear operation, we can then assume the OTF can be decomposed as:

$$\hat{X} = \hat{C_p} + \hat{\eta_p} + \hat{\eta_g} + \hat{b} \tag{21}$$

where $\hat{X}$ is the Fourier transform of X. We then assume the signal ground truth transforms into a perfect OTF with no background and DC term equal $\overline{C_p}$. The bias offset will only have a frequency component in the DC term equal to b. As the noise terms are spectrally white, they will have a constant power across all frequencies. This power can be found using Wiener-Khinchin theorem[2]:

$$\overline{S}_X = |\hat{X}|^2 \tag{22}$$

where $\overline{S}_X$ is the average power spectral density of X. We can find the average power of the Fourier transform of the noise terms:

$$\overline{S}_\eta = \sum_h \gamma_{eta}(h) e^{-2\pi i \nu h} \tag{23}$$

where $\gamma_{eta}(h)$ is the autocovariance of the noise term η, and ν is the frequency. As the noise is spectrally white, this only leads to the variance of the noise, and we get:

$$\overline{S}_\eta = \overline{S}_{\eta_p} + \overline{S}_{\eta_g} = C_p + \sigma_{RMS}^2 \tag{24}$$

$$|\hat{\eta}| = \sqrt{\overline{C_p} + \sigma_{RMS}^2} \tag{25}$$

as we don't know C_p, we can estimate it using the DC term of the OTF. As the DC term is composed of the signal and the bias offset, we get:

$$\overline{C_p} = DC_{\hat{X}} - b \tag{26}$$

where $DC_{\hat{X}}$ is the DC term of the OTF. Using this we can find the average spectral power of the background as:

$$|\hat{\eta}| = \sqrt{DC_{\hat{X}} - b + \sigma_{RMS}^2} \tag{27}$$

By subtracting this background level from the OTF, we define the cutoff frequency as the first zero-crossing of the resulting OTF. Then, assuming the OTF is an ellipsoid, we can find the PSF volume and are from the resulting resolution limits.

Supplementary figures

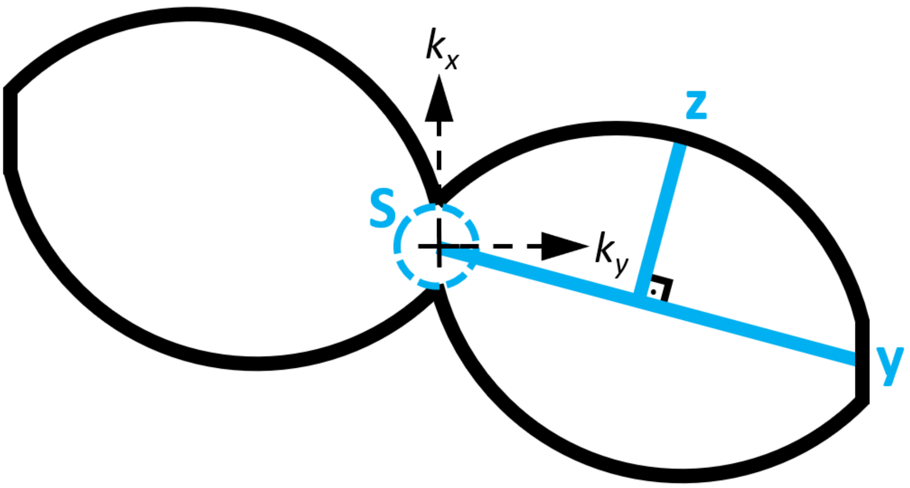

Supplementary Figure 1: Pictogram of OTF showing the sectioning thickness S, y resolution, and z resolution.

References

[1] M. Leutenegger, R. Rao, R. A. Leitgeb, and T. Lasser, "Fast focus field calculations," *Optics express*, vol. 14, no. 23, pp. 11 277–11 291, 2006.

[2] J. Y. Stein, *A computer science perspective*. Wiley, 2000.
